# Reduced Isocyanate Release Using a Waterproof, Resin-Based Cast Alternative Relative to Fiberglass Casts

**DOI:** 10.3390/toxics11121002

**Published:** 2023-12-08

**Authors:** Kristen Stefanescu, Claire L. Timlin, Ashley S. Moy, Grzegorz Zapotoczny

**Affiliations:** 1 Keck School of Medicine of the University of Southern California, 1975 Zonal Ave., Los Angeles, CA 90033, USA; kstefane@usc.edu; 2 Cast21, 1623 W Fulton St., Chicago, IL 60612, USA; claire.fourriverskennel@gmail.com; 3 Consortium for Technology & Innovation in Pediatrics, Lurie Children’s Hospital, 225 E Chicago Ave., Chicago, IL 60611, USA; gzapotoczny@luriechildrens.org

**Keywords:** orthopedic, cast, isocyanate

## Abstract

The effects of occupational isocyanate exposure range from asthma and contact dermatitis to neurotoxicity and cancer. Respiratory sensitization due to orthopedic cast application has been well documented. This study aims to compare the safety of standard-of-care fiberglass casts and a novel waterproof cast alternative by measuring the amount of isocyanate released during off-gassing over time. A 3D-printed arm simulator with comparable casing material amounts was placed in a sealed chamber. An isocyanate-sensing color-changing (SafeAir) tag was used to measure the levels of toxic exposure. Triplicate trials were conducted across all time periods (15 min, 1 h, and 24 h) and conditions. The bare arm simulator and freshly opened tags served as negative controls. Normalized pixel intensity indexes and isocyanate release estimates in ppb were derived from ImageJ-analyzed SafeAir tag photos. Fiberglass casts exhibited greater isocyanate release than both the waterproof alternative (*p* = 0.0002) and no-cast controls (*p* = 0.0006), particularly at 24 h. The waterproof alternative and no-cast control did not statistically differ (*p* = 0.1603). Therefore, the waterproof alternative released less isocyanate than the fiberglass casts. Waterproof cast alternatives may be safer than fiberglass by limiting medical professionals’ exposure to toxic isocyanates and, thus, decreasing their risk of suffering occupational asthma.

## 1. Introduction

**Figure 1:**
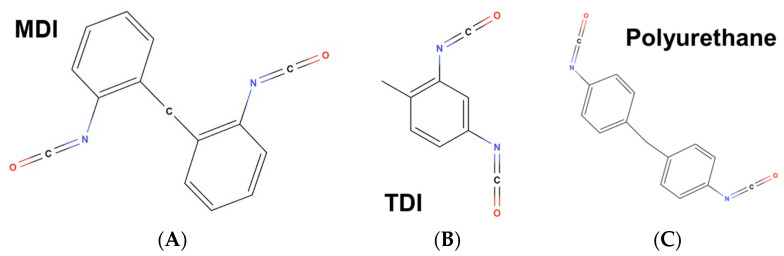
Representative figures of the following compounds: (**A**) methylene diphenyl diisocyanate (MDI), (**B**) toluene diisocyanate (TDI), and (**C**) polyurethane. The figures were created using MolView.

Isocyanates, including di- and polyisocyanates, are reactive compounds distinguished by their -N=C=O functional group, which is known to cause occupational asthma [1,2,3]. Among these chemicals, methylene diphenyl diisocyanate (MDI) and toluene diisocyanate (TDI) are some of the most commonly used isocyanates (see Figure 1A,B). Isocyanates are frequently found in polyurethane foams and flame retardants, and some isocyanate-containing compounds exhibit significant degradation in soil [3,4,5]. Isocyanates have likewise been found in a variety of biomedical devices, including orthopedic casting materials [4,6]. A variety of adverse health consequences beyond respiratory sensitization have been reported, including contact dermatitis, as well as neurotoxicity and cognitive impairment [1,7]. Furthermore, the National Toxicology Program of the Department of Health and Human Services published that TDIs are “anticipated to be human carcinogens”, necessitating additional investigation [1,8].

Despite the multitude of potential health consequences of isocyanate exposure, asthma remains the most frequently documented and thoroughly studied phenomenon, secondary to occupational contact. Upon inhalation, isocyanates may react with the proteins and other compounds of the epithelial lining fluid coating respiratory mucous membranes [9]. While the biophysical process via which respiratory sensitization subsequently occurs has not been fully elucidated in human or animal models, a number of murine models have exhibited increased IgE levels and cytokine release upon isocyanate exposure [10,11]. One study demonstrated significant murine airway epithelial injury through the induction of the pyroptosis mechanism of cell death in these tissues, while others have explored other biochemical cascades that may contribute to respiratory epithelial cell dysregulation and inflammation [12,13,14]. Human studies have likewise shown isocyanate-specific IgE antibodies in sensitized individuals and a variety of isocyanate–protein adducts, while others have identified potential genetic polymorphisms that predispose individuals to developing isocyanate-induced asthma, including genes involved in inflammatory processes, such as those for the major histocompatibility complexes I and II and TNFα [15,16,17,18]. It has been theorized that protein adducts, as well as isocyanate-specific antibodies, may induce immune response, although further study is needed to determine their relationship with respiratory sensitization [19,20]. The potential for cutaneous exposure to induce sensitization likewise demands further study [21,22,23].

Regardless of the mechanism used, the resultant asthmatic response poses a significant risk, particularly as a safe level of isocyanate exposure is difficult to determine, with estimates ranging from an average dose of 1 ppb or less up to 20 ppb [2,3,24,25,26,27]. Notably, subsequent asthmatic reactions in sensitized individuals have been documented after exposure to levels as low as 1 ppb [27]. Determining a safe level of aerosol exposure in particular is made more challenging by the unstable nature of isocyanate molecules and heterogenous size of their particles, as well as the sensitivity of testing methodologies required to identify low levels of exposure [2]. Given that methods for examining worker exposure by quantifying levels of isocyanate-specific antibodies or albumin adducts, while developed, may exhibit variability and are not in use, minimizing exposure is vital [28,29].

Although isocyanates are most frequently used in the production of surface coatings and adhesives, occupational exposure in healthcare settings has been reported [2]. Despite one study implying that the risk of inhalation or cutaneous exposure to isocyanates during orthopedic casting is low, multiple case reports of demonstrable isocyanate sensitization among orthopedic healthcare providers have been published [30]. In the earliest such report, an orthopedic surgeon was found to have isocyanate-induced asthma, as indicated via a positive “inhalation test” and confirmed via ELISA showing IgG specific to MDI and TDI [31]. Subsequent reports of four nurses across different facilities and countries demonstrated respiratory sensitization to MDI, as confirmed via bronchial provocation testing, resulting in asthmatic response with diminished forced expiratory volume (FEV1) in all cases [6,32,33]. Even in these instances of exposure to relatively low levels of isocyanate, estimated by the most recent case reports to be approximately 10 ppb, healthcare workers can experience asthma attacks due to their occupational exposure to these compounds [6]. As of the time of writing, no studies have examined the incidence of occupational asthma secondary to isocyanate exposure in a medical setting.

Despite rapid progress in orthopedic medical technologies over the past 20 years, traditional casts made of plaster or fiberglass have remained a mainstay for limb immobilization [34,35,36]. The fiberglass tape used in such casts is impregnated with polyurethane, thereby presenting health risks to clinical staff given the frequent occupational exposure to isocyanates (see Figure 1C) [37]. Moreover, traditional casting methodology is limited by its water-sensitive nature, leaving patients at risk of complications ranging from minor skin irritation to compartment syndrome should their cast become wet, requiring the reapplication of the cast or further treatment [38,39]. To address the limitations of traditional plaster and fiberglass casts, a novel waterproof cast alternative has been developed by Cast21. Whereas fiberglass casting methods require that rolls of fiberglass tape be applied over an affected body area, the waterproof cast alternative employs a closed system that does not directly expose the medical professional applying the product, or the patient, to isocyanate off-gassing materials. The waterproof cast alternative requires the application of a hollow immobilization net over the injured limb. The net is then filled through a single port with a fast-curing resin from an external pack. The resin subsequently hardens to provide necessary structure and stability to the cast alternative. Therefore, the waterproof cast alternative limits exposure to the essential and potentially isocyanate-laden resin. The purpose of this investigation is to demonstrate that the novel design of the waterproof cast alternative, which encompasses engineering safety controls, leads to lower isocyanate exposure than fiberglass casts. This may, in turn, reduce the risk of occupational asthma among healthcare workers who routinely use such cast alternatives.

## 2. Materials and Methods

### 2.1. Waterproof Alternative and Fiberglass Testing

**Figure 2:**
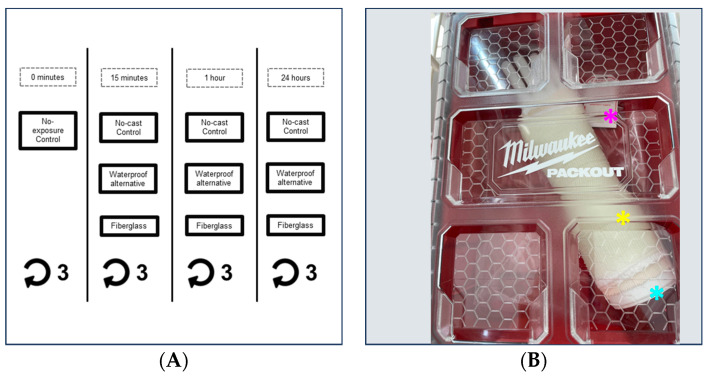
Isocyanate testing methodology and experimental setup. (**A**) Experimental paradigm of no-cast control, waterproof alternative, and fiberglass triplicate trials conducted for three distinct time periods of 15 min, 1 h, and 24 h. Additionally, the three no-exposure controls that represent a time period of 0 min are represented here. (**B**) Photo of the sample sealed chamber, arm simulator (cyan asterisk) with the fiberglass cast (yellow asterisk) applied, and SafeAir tag (magenta asterisk).

Each trial quantified the isocyanate released by comparable quantities of the waterproof alternative (Cast21 Short Arm Product; size XS-extra small) and fiberglass casts. All experiments were conducted at room temperature (20–24 °C) in a professional office setting. The comparable amount of fiberglass for each trial was determined by a skilled orthopedic casting technician, with fiberglass component quantities representing typical variations in casting product quantities used in a clinical setting. An average of 2.856 m (range of 2.75–3.47 m) of fiberglass casting material was used in each trial (see Supplementary Table S1). Isocyanate release by either the waterproof cast alternative or an average of 3.47 yards (range of 3.01–3.8 yards) of fiberglass casting material was evaluated after 15 min, 1 h, or 24 h within a sealed Milwaukee PACKOUT Compact Organizer (Product #48-22-8435) testing chamber, using a Morphix Technologies SafeAir TDI/MDI tag (Part #382001) (see Figure 2A). The product, be it the waterproof cast alternative or fiberglass, was applied to a reusable 3D printed arm simulator (Makerspace and Entrepreneur Center, Palatine, IL, USA), which was made of white polylactic acid filament of 1.73 mm in diameter using a purpose-built Voron 0 3D printer. The arm simulator and casting components were then placed in the clean testing chamber, which was lined with plastic lining, alongside a SafeAir tag (see Figure 2B). The testing chamber was then sealed for the duration of the trial. For the no-cast control trials, the above protocol was followed, but no product was applied to the arm simulator prior to placement in the testing chamber. Additionally, three SafeAir tags that were never placed in the testing chamber were evaluated immediately after they were unsealed to assure no pre-existing baseline isocyanate readings; these are considered the no-exposure control trials.

Appropriate measures were taken to limit the influence of each trial on that which followed it. A new plastic lining was placed in the testing chamber for each trial. The testing chamber and arm simulator were cleaned between each trial using a 70% ethanol solution and dried with a paper towel.

### 2.2. Image Collection

**Figure 3:**
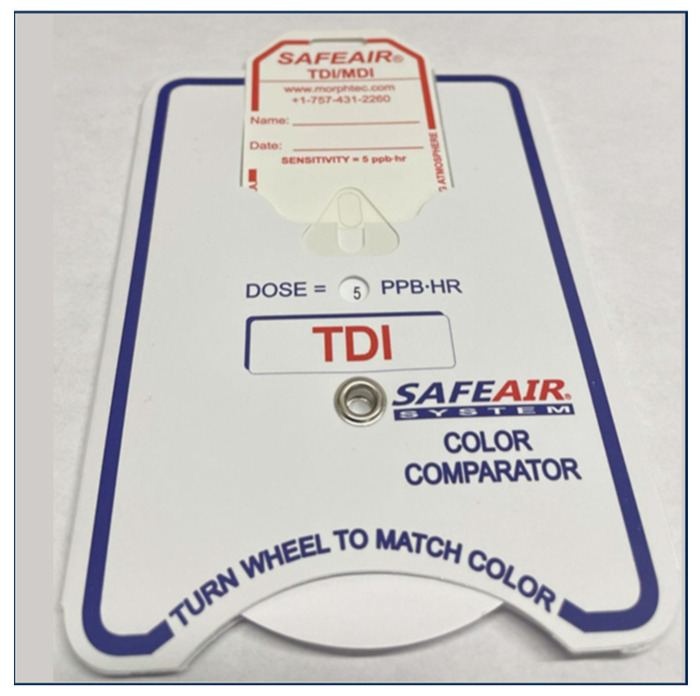
SafeAir tag inserted into the Color Comparator.

After each trial, the SafeAir TDI/MDI tag was placed in a SafeAir TDI Color Comparator (Part#: 383005) (Morphix Technologies, Virginia Beach, VA, USA). The Color Comparator was designed to allow the user to compare the coloration of the responsive part of the tag to the color present on the Color Comparator wheel, with each color representing a different estimated concentration of isocyanate (Figure 3). The SafeAir tag was color matched to the Color Comparator, and the tag and comparator were imaged using the camera on an iPhone 12 mini. Then, the Color Comparator wheel was spun such that one color unit above and below the presumed color match was also shown relative to the tag and photographed. Therefore, each trial resulted in three images: one color matched to the tag, one was such that the Color Comparator color was below the color matched unit, and one was above the color matched unit. The camera lens was cleaned between trials, and all tags were photographed head-on to minimize the impact of shadows on pixel intensities. All images were uploaded as .HEIC files of 2340 × 1080 pixel resolution at 476 ppi. All image files were subsequently converted to .JPG files prior to image analysis.

### 2.3. Image Analysis

**Figure 4:**
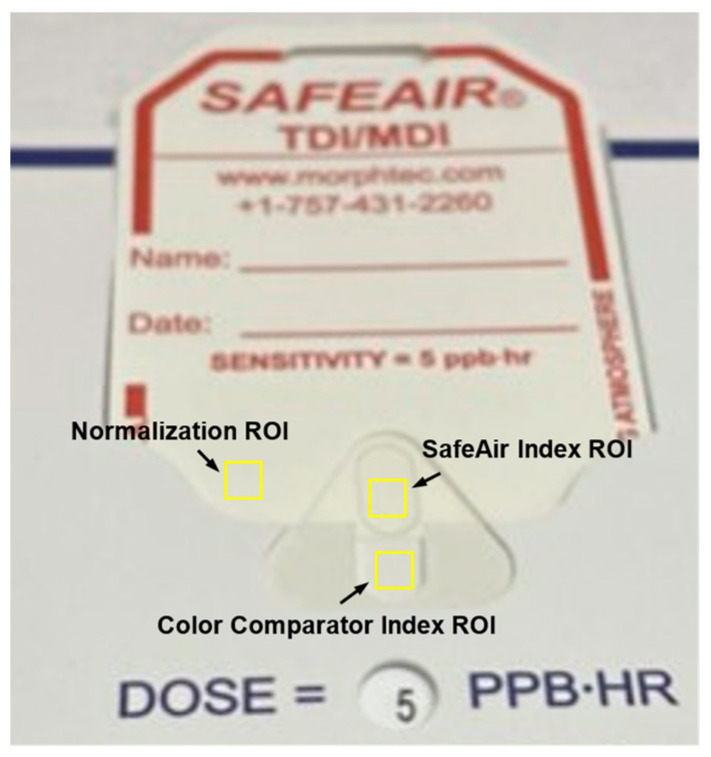
Screen capture demonstrating the location of the SafeAir Index ROI, the Color Comparator Index ROI, and the normalization value ROI overlaid on an image of a SafeAir tag inserted into the Color Comparator.

Each image was opened in ImageJ/Fiji software (updated on 9 March 2023) (version 2.9.0, National Institutes of Health, NY, USA), a program that can measure pixel intensities, wherein a large value reflects a brighter or lighter object. A 50 × 50 pixel square was used to sample all regions of interest (ROIs), as henceforth described. An ROI over the active, color-changing SafeAir tag component was sampled, and the median pixel intensity of all 2500 pixels in this ROI was recorded to serve as the SafeAir Index. ROIs were likewise sampled over the color matching component of the Color Comparator to serve as the Color Comparator Index, as was an inactive part of the SafeAir tag to serve as the Normalization value (Figure 4). Again, median values from these ROIs were recorded.

This process was repeated for each image. Once all three images for each trial were analyzed, the absolute value of the difference between the SafeAir Index and the Color Comparator Index was compared between the three trials. The trial with the lowest absolute difference, as described above, was deemed to be the true color match, and only its data were used in all subsequent statistical analyses. The estimated isocyanate release (ppb) displayed on the Color Comparator in the true color-matched image was transcribed. The normalized SafeAir Index from that same true color-matched image was calculated using the following formula (Figure 4):

$$\frac{\mathrm{SafeAir}\;\mathrm{Index}\;\mathrm{value}}{\mathrm{Normalized}\;\mathrm{value}}=\mathrm{normalized}\;\mathrm{SafeAir}\;\mathrm{Index}$$

Using the normalized SafeAir Index for comparisons of the SafeAir tag response to quantify the relative isocyanate concentration, the potential effect of variability in lighting conditions between images was minimized.

Both the estimated isocyanate release (ppb) and normalized SafeAir Index values were evaluated for best matching images in order to provide as nuanced an evaluation of relative isocyanate release as possible. As the Color Comparator estimates increased in a non-linear manner (from 0 to 5, then 7, then 10, then by 5 thereafter), it was considered most useful for a real-world setting to examine these estimated values exactly as provided by the tool likely to be used in practice. In contrast, the normalized SafeAir Index values were used as a proxy for more granular changes in isocyanate release by the casts tested, allowing greater scrutiny in comparing isocyanate release without assuming, altering, or otherwise obfuscating the values provided by the SafeAir tags; these normalized SafeAir Index values were a relative measure of isocyanate release.

### 2.4. Statistical Analysis

Data analysis was performed using SAS OnDemand software, Version 3.81, copyright © 2012–2020 (SAS Institute Inc., Cary, NC, USA). The SafeAir Index was analyzed using a repeated measures model (PROC MIXED) with fixed effects of time, cast, and cast by time interaction and cast within chamber as the repeated subject. The model was run using the time period as a categorical variable and sequential sum of squares, with the no-exposure control trials included as covariates. Differences between cast types and time periods were assessed with Tukey’s post hoc analysis. Estimated isocyanate ppb values were analyzed using the Kruskal–Wallis one-way analysis of variance between cast type for each time period and between time periods across cast types with pairwise two-sided Dwass, Steel, and Critchlow–Fligner (DSCF) multiple comparison analysis. Significant differences were defined as *p* < 0.05 for all statistical tests.

## 3. Results

### 3.1. Normalized SafeAir Index Values

**Figure 5:**
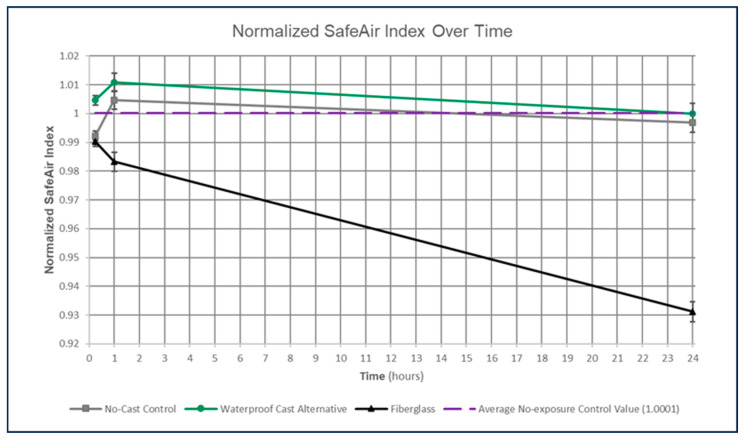
Graph of normalized SafeAir Index values over time, separated by group (no-cast control, waterproof alternative, and fiberglass), with lines connecting the average values that have standard deviation error bars, as well as the average no-exposure control normalized SafeAir Index value of 1.0001. Lower normalized SafeAir Index values reflect an increase in isocyanate release. Standard deviation values were identical by time period: 0.00167 for 15-min, 0.00322 for 1-h, and 0.00347 for 24-h time periods. Waterproof alternative exhibited less isocyanate release than fiberglass casts for each normalized SafeAir Index.

Isocyanate release over time was lower for the waterproof alternative relative to fiberglass casts, as evaluated using the normalized SafeAir Index values. The lower the isocyanate release, the less the response and, therefore, the lighter the color on the reactive region of the SafeAir tag, resulting in a higher pixel intensity and, thus, higher SafeAir Index-to-normalized value ratio. Conversely, lower normalized SafeAir Index values denoted greater isocyanate release. Visually, there was no notable difference between the average normalized SafeAir Index value derived from the freshly opened, no-exposure controls and the normalized SafeAir index values obtained from the no-cast controls or the waterproof alternative (see Figure 5). In contrast, the fiberglass normalized SafeAir index values for the 24-h time period are significantly lower than those derived from the no-exposure SafeAir tags (see Figure 5).

Across all time periods, the no-cast controls and waterproof alternative exhibited similar, not statistically different normalized SafeAir Index values (*p* = 0.1603; see Table 1). In contrast, fiberglass normalized SafeAir Index values were much lower than both no-cast controls (*p* = 0.0006) and the waterproof alternative (*p* = 0.0002; see Table 1), indicating greater isocyanate release. Therefore, the waterproof alternative released less isocyanate overall than the fiberglass cast. 

To determine whether this finding could be explained by time alone independent of cast type, normalized SafeAir Index values were compared between time periods without separating by cast type. There was no statistically significant difference in normalized SafeAir Index values between 15-min and 1-h time periods (*p* = 0.0774; see Table 2). However, normalized SafeAir Index values obtained from 24-h tests were significantly different from those obtained from 1-h (*p* = 0.0003) and 15-min (*p* = 0.0002; see Table 2) tests. Therefore, as indicated visually by plotting normalized SafeAir Index values over time, the greatest difference between groups occurred at 24 h, with fiberglass exhibiting much larger isocyanate release than the no-cast controls and waterproof alternative (see Figure 5).

In evaluating all statistical assessments of normalized SafeAir Index values as a proxy for isocyanate release, fiberglass casts released more isocyanate overall, with the greatest increase in isocyanate release demonstrated at 24 h relative to the waterproof alternative and no-cast controls (see Figure 5). In contrast, the waterproof alternative released isocyanate at levels statistically indistinguishable from the no-cast controls and quantities significantly lower than the fiberglass cast.

### 3.2. Estimated Isocyanate Release in PPB

**Figure 6:**
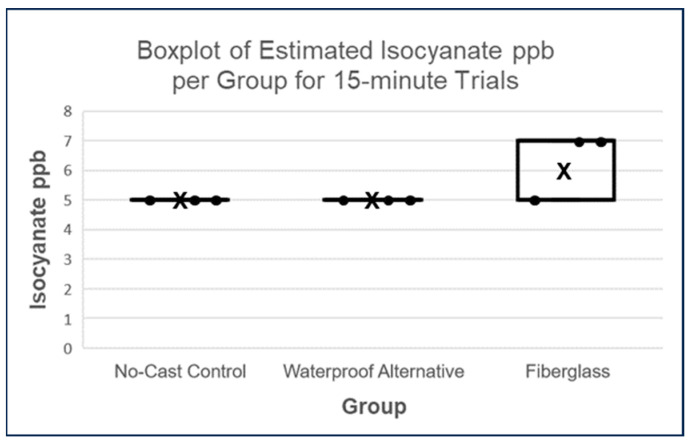
Boxplots of estimated isocyanate ppb scores per group for 15-min trials, where each datapoint is represented by a dot and each cross denotes the mean of a given set of values. The Kruskal–Wallis test resulted in a *p*-value > 0.05 (*p*-value = 0.1017), indicating no significant difference between groups.

Consistent with the normalized SafeAir Index results, the isocyanate ppb release estimates also demonstrated less isocyanate release by the waterproof alternative relative to fiberglass casts, albeit after 1 h and 24 h. At 15 min, estimated isocyanate ppb values did not significantly differ between groups (Kruskal–Wallis test, *p* = 0.1017; see Figure 6). 

**Figure 7:**
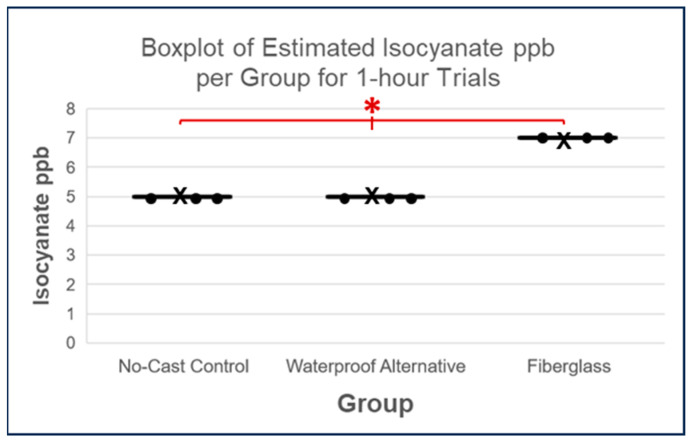
Boxplots of estimated isocyanate ppb scores per group for 1-h trials where each datapoint is represented by a dot and each cross denotes the mean of a given set of values. The Kruskal–Wallis test resulted in a *p*-value < 0.05 (*p*-value = 0.0183), indicating a significant difference between all groups, as symbolized by the red asterisk.

However, after 1 h, values differed significantly based on the Kruskal–Wallis test (*p* = 0.0183; see Figure 7). Post hoc analysis via DSCF showed trending differences in estimated isocyanate ppb between the fiberglass cast and waterproof alternative (*p* = 0.0653) and control (*p* = 0.0653; see Table 3). Although these results were not statistically significant, given the statistically significant differences shown previously via an analysis of normalized SafeAir Index values, isocyanate release estimates may not have reached statistical significance due to the more discrete nature of isocyanate release quantification employed in this methodology. Nevertheless, this DSCF comparison of no-cast controls to waterproof alternative yielded a *p*-value of 1, indicating no difference between these groups, which is consistent with the analysis of normalized SafeAir tag values.

**Figure 8:**
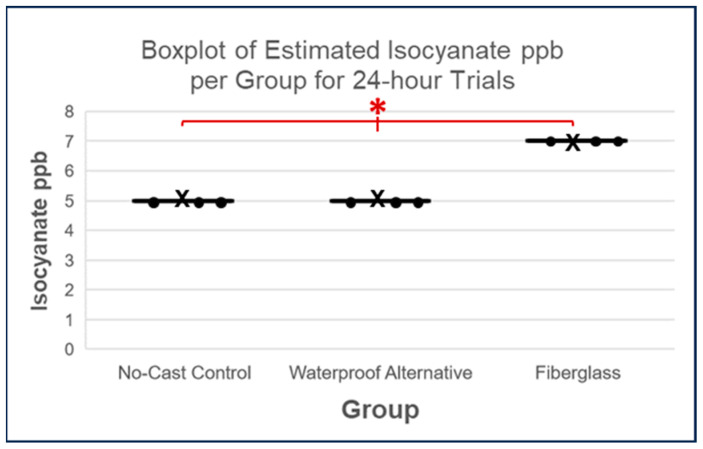
Boxplots of estimated isocyanate ppb scores per group for 24-h trials where each datapoint is represented by a dot and each cross denotes the mean of a given set of values. The Kruskal–Wallis test resulted in a *p*-value < 0.05 (*p*-value = 0.0183), indicating a significant difference between all groups, as symbolized by the red asterisk.

Similar results were observed after 24 h, with the elevated estimated isocyanate ppb from the fiberglass casts (Kruskal–Wallis test, *p* = 0.0183; see Figure 8). Again, the DSCF analysis found that the fiberglass cast values were not statistically significant despite low *p*-values in comparisons of fiberglass cast with the waterproof alternative (*p* = 0.0653) and no-cast controls (*p* = 0.0653; see Table 4). As was the case when comparing values for 1-h trials, the DSCF comparison of values of 24-h control trials with the waterproof alternative trials yielded a *p*-value of 1. 

**Figure 9:**
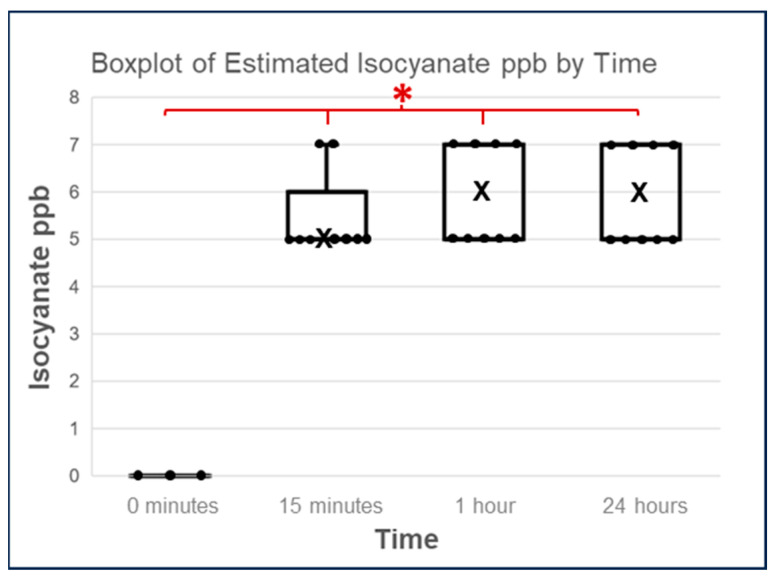
Boxplots of estimated isocyanate ppb scores per time period, where each datapoint is represented by a dot and each cross denotes the mean of a given set of values. The Kruskal–Wallis test resulted in a *p*-value < 0.05 (*p*-value = 0.0116), indicating a significant difference between all time periods, as symbolized by the red asterisk.

Estimated isocyanate ppb values were also examined to determine whether time alone, regardless of cast group, influenced the relative isocyanate release using this additional measure. To evaluate the influence of time alone on estimated isocyanate ppb release, analysis of estimated isocyanate ppb values by time was run and showed significant differences overall (Kruskal–Wallis test, *p* = 0.0116; see Figure 9). Comparisons of no-exposure control values with values from all other time periods demonstrated statistically significant differences (*p* < 0.05), whereas comparisons of estimated ppb values from all other time periods (15 min, 1 h, and 24 h) were not statistically significant (*p* > 0.9, see Table 5). In particular, the DSCF comparison of 1-h with 24-h estimated ppb values yielded a *p*-value of 1. These results are as expected, as the no-exposure control values were all 0 ppb, whereas estimated isocyanate ppb increased thereafter for all time periods and exhibited identical distributions for the 1-h and 24-h time periods (see Figure 9). Taken together, these findings imply that time alone did not significantly influence isocyanate release for all groups, as the only statistically significant differences were found in comparisons between no-exposure controls and all other time periods. 

Therefore, the analysis of estimated isocyanate release in ppb implied once again that the waterproof alternative released less isocyanate than the fiberglass cast after 1 h and 24 h (see Figure 6 and Figure 7). Although this differs from the normalized SafeAir Index value comparisons, which indicated a greater difference in isocyanate release between these cast types only after 24 h (see Figure 5), it nevertheless provides additional evidence that the waterproof alternative releases less isocyanate and implies that this difference, even when using more discrete isocyanate release estimates, occurs due to a difference in cast type rather than an effect of time alone (see Figure 9).

## 4. Discussion

The significant health risk posed by occupational isocyanate exposure is made more troubling by the lack of viable biomarkers for accurate exposure assessment, thereby necessitating that exposure be minimized altogether. Although studies have suggested using TDI-specific serum IgE or IgG antibodies to monitor exposure or predict the development of occupational asthma, establishing standardized antibody ranges has proved challenging [16]. Given the limited half-life of IgE antibodies, limited temporal presence of IgG antibodies after exposure, and variability in individual responses, reliance on antibodies as markers of exposure or disease may not be feasible [28]. While advancements in urinalysis and epigenome sequencing may provide future means of performing routine isocyanate exposure monitoring, no such method is currently widely available [40,41]. Difficulties in monitoring occupational exposure necessitate undertaking efforts to minimize isocyanate exposure. 

The present study focuses on the development and trial of isocyanate exposure methodologies that are cost effective and practical to use in an orthopedic setting. As patient exposure to isocyanates secondary to casting is brief and typically non-repetitive, this investigation grounds its purpose in promoting orthopedic healthcare provider and technician safety. The design of the Cast21 waterproof cast alternative was theorized to minimize isocyanate exposure due to the isolation of the isocyanate-containing resin within sealed bags prior to application and inside of the hollow immobilization net upon the device’s application.

To this end, the present study’s experiments helped provide insight and address two hypotheses. Firstly, the total amount of isocyanate released from each casting product over a 24-h period was examined. Using the SafeAir tag’s reactive color-changing component and matching it to the Color Comparator by eye (as per the product instructions), we found that the measurements from the waterproof cast alternative and the no-cast control were identical (approximately 5 ppb), most likely arising from a background read caused by the limitations in the tag’s sensitivity. This indicates that the waterproof cast successfully contained and minimized release of the toxic chemical. As a positive control, a traditional fiberglass cast was used, and the 24-h tests revealed that approximately 7 ppb isocyanate were released. The Kruskal–Wallis test confirmed that there was a significant (*p* = 0.0183) change in isocyanate release across the experimental conditions for the 1-h and 24-h timepoints. Subsequent DSCF statistical testing was used to perform pairwise comparisons to identify which group(s) were responsible for the statistically significant difference implied by the Kruskal–Wallis test results. However, DSCF tests yielded *p*-values of 0.0653 for comparisons of the waterproof alternative with fiberglass and fiberglass with no-cast controls. In contrast, comparisons of the waterproof alternative with no-cast controls by DSCF yielded *p*-values of 1, strongly indicating that there is no difference between these two groups. The lack of a post hoc *p*-value below the widely accepted threshold of significance (*p* < 0.05) for other comparisons was likely due to the small sample size, and an increase in the number of measurements could alleviate this issue in future studies. Likewise, the level of exposure from just one cast may not be sufficient for robust detection using this methodology. The SafeAir tag minimal detection level is 5 ppb, and the test resolution is low (detecting only 0, 5, 7, 10, and so on by 5 ppbs at a time, with a maximum of 140 ppb). Additionally, even the freshly opened tags that were not exposed to any isocyanate amounts can be misinterpreted by an observer and estimated as showing 5 ppb instead of zero. Nevertheless, all available data indicate that the overall difference between groups at these times was most likely due to lower waterproof alternative isocyanate release relative to fiberglass casts. Therefore, the unique design of the waterproof cast alternative alleviates the issue of off-gassing and limits the exposure to isocyanates when measured via an off-the shelf, simple, and widely available test. 

To improve the robustness of SafeAir tag testing and overcome its limitation on the isocyanate level detection, rather than matching the tag and comparator by eye, we employed image analysis software to confirm the best SafeAir color-changing reactive component to Color Comparator standard match, as well as compare relative SafeAir color-change intensity between tags. This allowed us to improve the isocyanate release estimates by deriving a normalized SafeAir Index used for the aforementioned comparison between tags. In turn, these results allowed us to greatly improve the confidence of our earlier observations and confirm significant differences between the fiberglass cast and the no-cast control across all time periods (Tukey’s post hoc comparison, *p* = 0.0006) and between the fiberglass cast and the waterproof alternative across all time periods (*p* = 0.0002). This methodology, although requiring more intensive data analysis, can be successfully employed when evaluating the isocyanate release with the SafeAir tags in the environment where the analysis by eye is challenging or greater accuracy is required. 

Our experiments and the investigation of isocyanates as a root cause of occupational asthma were designed to simulate varying degrees of exposure depending on a stakeholder. The 15-min test was used to establish a minimum amount of isocyanate exposure, as this is the minimum time period for which the SafeAir tag is approved. Thereafter, the 1-h exposure could be the equivalent of what the patient experiences during the cast application process and likely approaches the time period required for the cast to set such that it may bear weight, while the 24-h test in our experiments intends to simulate the repeated exposure of the medical personnel responsible for casting to isocyanates [37]. This allowed us to gain a unique insight into isocyanate release from a single cast over various time periods. Given the aforementioned limitations of SafeAir tag technology, the 15-min tests for the no-cast control and waterproof cast alternative may not have reached the minimum 5 ppb exposure, despite being visually more likely to be similar to the 5 ppb marker on the Color Comparator. However, for the fiberglass casts, two out of three SafeAir tags showed 7 ppb isocyanate release. This could indicate that as little as 15 min of exposure to traditional fiberglass casting material could deliver substantial amounts of isocyanates to the patient or practitioner. For both the 1-h and 24-h trials, all fiberglass reads were at the levels of 7 ppb, whereas the SafeAir tag indicators showed 5 ppb for both the waterproof alternative and no-cast control. Given that the no-cast control tests did not have any casting material to deliver harmful isocyanates, it is likely that the 5 ppb readings reflected background noise rather than a bone-fide isocyanate exposure, again demonstrating a limitation of the SafeAir tag technology. The potential exposure is far greater and undoubtedly significant, despite the low n values for the 24-h time period. 

Taken together, the analysis of isocyanate release over time, as exemplified by the fiberglass cast readings, indicates that the longer exposure time, the higher dose of isocyanates that may be delivered to the human body. This is most likely a risk factor for medical staff that work in an environment where repeated and continuous exposure is likely. The volumes of patients might differ between facilities and season, with the peak during winter months with lower temperatures; however, numerous casting procedures typically occur in a single room each day [42]. Our pilot experiments conducted in a controlled environment provide initial evidence that may explain the earlier literature reports of isocyanate-induced asthma in healthcare personnel. Although beyond the scope of this project, in future studies, SafeAir tags could be placed in casting rooms and doctor’s offices to monitor isocyanate exposure and provide further insight into isocyanate release on timescales that encompass days or weeks rather than minutes and hours. Nevertheless, the choice of the casting material is critical and antiquated technologies like fiberglass casts do not provide any safety controls or mechanical barriers for the toxic gasses being released as a byproduct of the hardening of the casting material. Novel products like the waterproof cast alternative offer an effective solution not only for patients who are able to fully participate in the daily activities without the fear of getting the cast wet but also for the healthcare workers who no longer need to be exposed to toxic off-gassing, which, in turn, can minimize their risk of suffering occupational asthma.

## 5. Conclusions

Isocyanates pose significant health risks, the most thoroughly documented and investigated being the development of occupational asthma [1,3,20]. There is significant variability in research findings regarding maximum safe occupational exposure, and once sensitization occurs, exposure to much lower levels of isocyanates can induce an asthmatic response [3,19,27]. Despite healthcare provider exposure to very low levels of isocyanates, MDI and TDI off-gassing by orthopedic casts has been shown to cause respiratory sensitization among healthcare professionals responsible for cast placement and removal [6,30,31,32,33]. Therefore, making every effort to reduce and limit healthcare exposure to these compounds is essential.

The production of cast alternatives, like the waterproof device developed using Cast21, should minimize technician and patient exposure to isocyanates. Further studies examining isocyanate release by such waterproof alternatives and/or custom 3D-printed braces and splints in lieu of traditional casts may demonstrate their ability to reduce healthcare worker exposure to such compounds, although their ability to promote healing after the same injuries as traditional casts must be verified [43,44,45]. To the best of our knowledge, this investigation is the first attempt to characterize isocyanate off-gassing by casting alternatives. The evaluation of the Cast21 waterproof alternative found that the waterproof alternative released less (if any) isocyanate compared to similarly sized fiberglass casts. The fact that two methods of assessing isocyanate release demonstrated similar isocyanate off-gassing compared to the waterproof alternative relative to no-case controls strongly indicates how the unique design of the cast limits toxic isocyanate exposure. Therefore, the waterproof cast alternative evaluated in this study may lower the health risk inherent in cast application for orthopedic technicians.

## Figures and Tables

**Table 1:** Tukey’s Post Hoc Test for Cast Types.

Comparison	Adjusted *p*-Value
No-Cast Control vs. Waterproof Alternative	0.1603
No-Cast Control vs. Fiberglass	0.0006
Waterproof Alternative vs. Fiberglass	0.0002

**Table 2:** Tukey’s Post Hoc Test for Time Periods.

Comparison	Adjusted *p*-Value
15 min vs. 1 h	0.0774
1 h vs. 24 h	0.0003
15 min vs. 24 h	0.0002

**Table 3:** DSCF Post Hoc Test for 1-h Trials.

Comparison	Adjusted *p*-Value
No-Cast Control vs. Waterproof Alternative	1.0000
No-Cast Control vs. Fiberglass	0.0653
Waterproof Alternative vs. Fiberglass	0.0653

**Table 4:** DSCF Post Hoc Test for 24-h Trials.

Comparison	Adjusted *p*-Value
No-Cast Control vs. Waterproof Alternative	1.0000
No-Cast Control vs. Fiberglass	0.0653
Waterproof Alternative vs. Fiberglass	0.0653

**Table 5:** DSCF Post Hoc Test for Time Periods.

Comparison	Adjusted *p*-Value
0 vs. 15 min	0.0252
0 vs. 1 h	0.0342
1 h vs. 24 h	0.0342
15 min vs. 1 h	0.9564
1 h vs. 24 h	0.9564
15 min vs. 24 h	1.0000

## Data Availability

The data presented in this study are available on request from the corresponding author.

## Supplementary Materials

The following supporting information can be downloaded via this link: https://www.mdpi.com/article/10.3390/toxics11121002/s1, Table S1: Fiberglass Casting Materials Used.
