# Supplementary figures and images for: Reduced Isocyanate Release Using a Waterproof, Resin-Based Cast Alternative Relative to Fiberglass Casts
Source: Toxics. 2023 Dec 8;11(12):1002. doi: 10.3390/toxics11121002 (PMC10747184; doi:10.3390/toxics11121002)

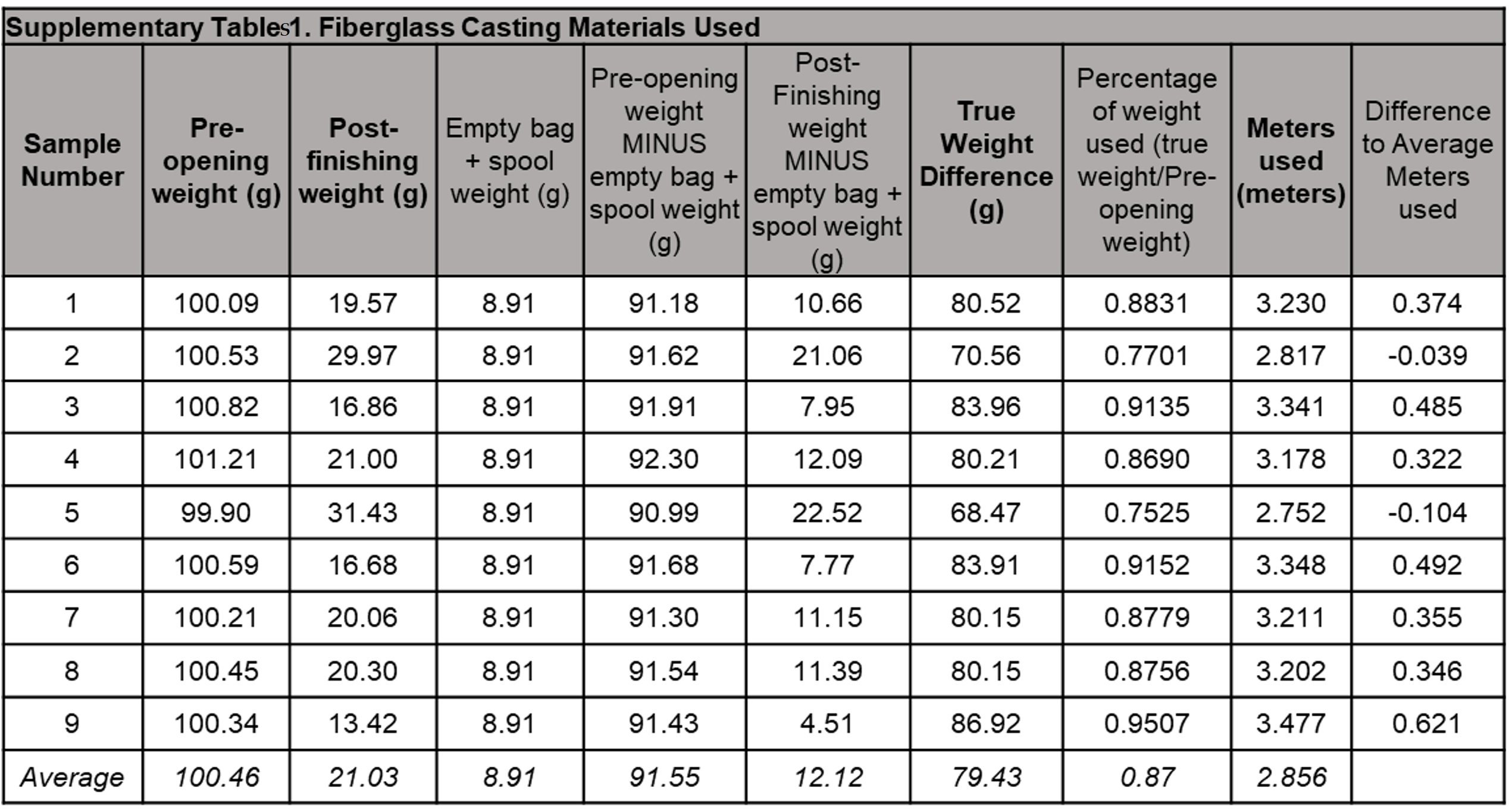

Supplement: Supplementary file 1 [file toxics-11-01002-s001.zip › toxics-2738573-supplementary.png]
